# Conjugated Metabolites of Hydroxytyrosol and Tyrosol Contribute to the Maintenance of Nitric Oxide Balance in Human Aortic Endothelial Cells at Physiologically Relevant Concentrations

**DOI:** 10.3390/molecules26247480

**Published:** 2021-12-10

**Authors:** Gabriele Serreli, Melanie Le Sayec, Camilla Diotallevi, Alice Teissier, Monica Deiana, Giulia Corona

**Affiliations:** 1 School of Life and Health Sciences, University of Roehampton, London SW15 4JD, UK; gabriele.serreli@unica.it; 2 Department of Biomedical Sciences, University of Cagliari, Monserrato, 09042 Cagliari, Italy; mdeiana@unica.it

**Keywords:** olive oil, conjugate metabolites, sulfate, glucuronide, hydroxytyrosol, tyrosol, phenolic acids, superoxide, nitric oxide

## Abstract

Nitric oxide (NO) is an important signaling molecule involved in many pathophysiological processes. NO mediates vasodilation and blood flow in the arteries, and its action contributes to maintaining vascular homeostasis by inhibiting vascular smooth muscle contraction and growth, platelet aggregation, and leukocyte adhesion to the endothelium. Dietary antioxidants and their metabolites have been found to be directly and/or indirectly involved in the modulation of the intracellular signals that lead to the production of NO. The purpose of this study was to investigate the contribution of conjugated metabolites of hydroxytyrosol (HT) and tyrosol (TYR) to the release of NO at the vascular level, and the related mechanism of action, in comparison to their parental forms. Experiments were performed in human aortic endothelial cells (HAEC) to evaluate the superoxide production, the release of NO and production of cyclic guanosine monophosphate (cGMP), the activation of serine/threonine-protein kinase 1 (Akt1), and the activation state of endothelial nitric oxide synthase (eNOS). It was observed that the tested phenolic compounds enhanced NO and cGMP concentration, inhibiting its depletion caused by superoxide overproduction. Moreover, some of them enhanced the activation of Akt (TYR, HT metabolites) and eNOS (HT, HVA, TYR-S, HT-3S). Overall, the obtained data showed that these compounds promote NO production and availability, suggesting that HT and TYR conjugated metabolites may contribute to the effects of parental extra virgin olive oil (EVOO) phenolics in the prevention of cardiovascular diseases.

## 1. Introduction

**Figure 1:**
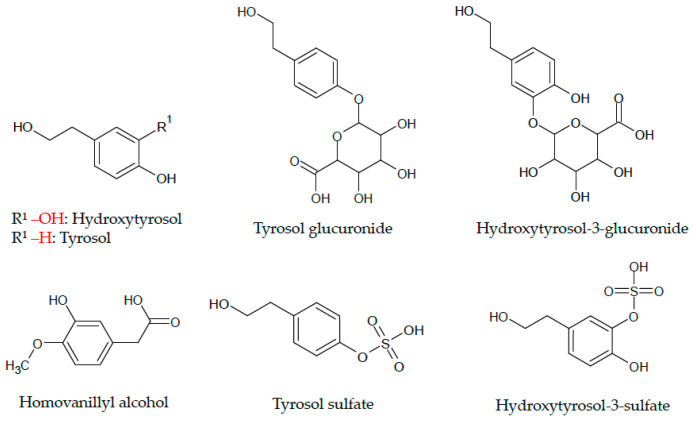
Chemical structure of all EVOO phenolics and metabolites tested in the study.

Nitric oxide (NO) is a key modulator of vascular function, and an imbalance in endothelium-derived NO can underlie the pathogenesis of vascular impairments, inducing the progression to hypertension, atherosclerosis, and vasospasm, affecting blood flow in the vessels [1]. The basal release of NO is indeed important in order to maintain the basal vascular tone, whereas the inhibition of NO synthesis in the vascular cells can lead to elevated blood pressure [2,3,4]. Therefore, injuries or dysfunctional endothelial cells with a loss of endothelium-derived NO are major critical factors in the pathogenesis of cardiovascular diseases (CVDs) [1,4]. Indeed, in CVDs, the downregulation of NO production plays a pivotal role in the impairment of the endothelial function [4] and promotes atherosclerosis through various mechanisms [5]. In addition, NO availability can be reduced via oxidative mechanisms, for example through the reaction with superoxide anions [6,7]. Vascular nicotinamide adenine dinucleotide phosphate (NADPH) oxidase is a major source of superoxide production in endothelial cells and is upregulated in the presence of cardiovascular risk factors [4,6]. Additionally, endothelial nitric oxide synthase (eNOS) uncoupling can lead to endothelial dysfunction, and the upregulation of eNOS activity through the activation of serine/threonine-protein kinase 1 (Akt1) contributes to maintaining or restoring a healthy vascular function [8]. Evidence from the literature suggests that olive oil phenolics play a role in the maintenance of endothelial function through oxidative-stress-related mechanisms, including a higher NO bioavailability [9]. Hydroxytyrosol (HT) and tyrosol (TYR) are two bioactive phenolic acids abundant in extra virgin olive oil (EVOO) and have been the subject of a range of investigations elucidating their role on the health benefits associated with the regular consumption of EVOO [10,11,12], including the prevention of cardiovascular diseases and the maintenance of healthy vascular function [3,9,13]. A sub-study of the PREDIMED trial, for example, showed that EVOO consumption resulted in lower circulating inflammatory biomarkers and cardiovascular risk factors, suggesting a dose-dependent anti-inflammatory effect of EVOO polyphenols [13]. In addition, an independent association between high urinary excretion of homovanillyl alcohol (HVA) and a lower risk of CVD and total mortality was observed in elderly individuals [14]. Furthermore, randomized controlled trials confirm that the acute consumption of HT-rich olive leaf extracts positively modulates vascular function in vivo via the actions of absorbed phenolic metabolites, highlighting the potential contribution of olive oil phenolics metabolites to the benefits on vascular function [15]. In EVOO, HT and TYR are present predominantly as secoiridoid derivatives, such as oleuropein and ligstroside aglycone derivatives [16]. After ingestion, secoiridoids aglycones can be hydrolyzed in the acidic gastric environment, leading to a significant increase in free forms (HT and TYR), which can be absorbed in the upper part of the gastrointestinal tract, predominantly in the small intestine [16]. Small-intestinal absorption occurs predominantly through passive diffusion. Both HT and TYR can then be further biotransformed in the gut and in the liver to produce their glucuronide- and sulfate-conjugated forms [16,17]. Due to the high metabolism of parental forms, metabolic products such as glucuronide and sulfate conjugates are the predominant bioavailable forms. In addition, HT also undergoes O-methylation to produce significant amounts of HVA, which can also be absorbed [16,17]. Overall, the available literature shows that HT, TYR, and metabolic derivatives (methylated, sulfated, glucuronidated forms) are bioavailable forms present in the circulation after EVOO consumption [16,17]. HT and TYR health benefits are derived from their redox properties as antioxidants (in particular HT, an *o*-diphenol); thus, their mechanisms of action go beyond the direct free radical scavenging properties [18,19,20]. Furthermore, the significant amounts of metabolic forms of HT and TYR found in the circulation raise the interest in the contribution of such forms to the health benefits in vivo [21,22,23,24]. Indeed, recent studies have started to take into great consideration the bioactivity and contribution to the beneficial health effects of the methylated, sulfated, and glucuronidated forms of HT and TYR [21,25,26,27,28]. Within this context, the aim of this study was to evaluate the potential modulation of NO balance by the bioavailable conjugated metabolites of HT and TYR in endothelial cells, in comparison to their parental forms. The chemical structure of all compounds tested is shown in Figure 1.

Human aortic endothelial cells (HAEC) were exposed to the compounds of interest within a physiologically relevant concentration range (1 µM) to assess the modulation of NO levels, cyclic guanosine monophosphate (cGMP), superoxide production, and Akt1/eNOS activation. The effects of the tested compounds were compared to those of apocynin, a known inhibitor of NADPH oxidase enzyme, and L-Nω-nitro-arginine (L-NNA), a competitive inhibitor of eNOS derived from N(ω)-nitro-L-arginine methyl ester (L-NAME) hydrolysis, for all NO-related assays.

## 2. Materials and Methods

### 2.1. Chemicals

L-arginine, apocynin, sodium nitrite, sodium nitrate, 3-(4,5-dimethylthiazol-2-yl)-2,5-diphenyltetrazolium bromide (MTT), Bradford reagent, cytochrome c from bovine heart, sodium dodecyl sulfate, superoxide dismutase from bovine erythrocytes, L-Nω-nitro-arginine (L-NNA), phosSTOP, cOmpleteMini, HT, TYR, and HVA were purchased from Sigma Aldrich (Gillingham, UK). Hydroxytyrosol-3-glucuronide (HT-3G), hydroxytyrosol-3-sulfate (HT-3S), tyrosol-glucuronide (TYR-G), and tyrosol-sulfate (TYR-S) were obtained from Toronto Research Chemicals Inc. (North York, ON, Canada).

### 2.2. Materials for Cell Culture

Human Aortic Endothelial Cells (HAEC), EBM-2 medium with and without phenol red, the BulletKit™—basal medium and SingleQuots™ and ReagentPack Subculture Reagents with 70 trypsin/EDTA, trypsin neutralizer solution (TNS), and 4-(2-hydroxyethyl)-1-piperazineethanesulfonic acid (HEPES) solutions were obtained from Lonza (Basel, Switzerland).

### 2.3. Maintenance of Cell Culture

HAEC cells were used at Passages 4–8 and grown in T75 flasks in EBM-2 media supplemented with 2% FBS, 0.2% heparin, 0.2% hydrocortisone, 0.2% human fibroblast growth factor basic (hFGFb), 0.2% human vascular endothelial growth factor (hVEGF), 0.2% long R3-IGF-1 (analog of human insulin-like growth factor-1), 0.2% ascorbic acid, 0.2% human epidermal growth factor (hEGF), and 0.2% GA 1000 (gentamicin sulfate) at 37 °C in a 5% CO_2_ humidified atmosphere. The subcultures were prepared by removing the cells with trypsin solution at 1% after washing with HEPES and then seeded into 6-well and 96-well plates for different experiments. Growth medium was replaced the day after seeding and every 2–3 days afterwards.

### 2.4. MTT Viability Test

The MTT assay [29] was performed using HAEC cells in order to evaluate any cytotoxic activity of the tested compounds. Cells were seeded in 96-well plates (5 × 10^4^ cells/mL; 100 µL/well) and, when confluent, were exposed to various concentrations of the compounds (1–10 µM, in serum-free medium), or an equivalent volume of MeOH for the controls, and incubated for 24 h. Afterwards, the medium was removed, and 100 µL of MTT solution (5 mg/mL of HBSS) was added and left for 4 h at 37 °C. The medium was then aspirated, 100 µL of DMSO was added to each well, and the absorbance was read at 570 nm by using a Multiskan Ex microplate reader (Thermo Scientific). Data were converted to % of cell viability as follows: % cell viability = Abs sample/Abs control × 100.

### 2.5. NO Analysis by DAF2-DA Fluorescence

NO analysis was performed in exposed cells using the DAF-2 diacetate (DAF2-DA) fluorescent probe [30] as previously described [31]. Briefly, HAEC cells were seeded in 96-well plates (5 × 10^4^ cells/mL; 100 µL/well) and, when confluent, were exposed to apocynin (100 µM), L-NNA (100 µM), the tested compounds (1 µM), or vehicle (MeOH) for 24 h. After the incubation time, a solution of DAF2-DA was added to the wells (0.5 µM final concentration, in HBSS) and kept for 4 h at 37 °C. The fluorescence signal was quantified on an FLx800 microplate reader (Biotek) at excitation and emission wavelengths of 485 and 528 nm, respectively. The fluorescence intensities were corrected by subtracting the nonspecific fluorescence in wells without the addition of DAF-2DA and in wells without cells.

### 2.6. Measurement of cGMP and Nitrites

In order to evaluate nitrite levels and cGMP production [32], HAEC cells were grown in 6-well plates and exposed to apocynin (100 µM), L-NNA (100 µM), the tested compounds (1 µM), or vehicle (MeOH) in phenol-free EBM-2 medium. After 24 h, the supernatants were collected and assayed for nitrite quantification by using the Nitrate/Nitrite Fluorometric Assay Kit (Cayman Chemicals, Ann Arbor, MI, USA). Afterwards, the cells were treated with 300 μL of HCl 0.1 μM and, after 15 min, were scraped and centrifuged in small Eppendorf tubes to collect supernatants. cGMP content was assessed using the Cyclic GMP competitive ELISA Kit (Cayman Chemical, MI, USA), following the manufacturer’s protocol.

### 2.7. Superoxide Release from Intact Cells

The evaluation of superoxide production was performed using a ferricytochrome c reduction method similar to the one used by Steffen et al. [33], as previously described [31]. Briefly, HAEC cells grown in 6-well plates were exposed to apocynin (100 µM), L-NNA (100 µM), the tested compounds (1 µM), or vehicle (MeOH) for 24 h. After the incubation time, cells were shortly washed with 50 mM phosphate buffer, pH 7.4, and exposed to 40 µmol/L ferricytochrome c in HEPES-buffered isotonic salt medium. Reduction of ferricytochrome c was measured in the supernatant at 550 nm (e = 21.1 mM^−1^ cm^−1^). Specificity of the assay for superoxide was ascertained by coincubation with superoxide dismutase (SOD; 200 U/mL). Superoxide release was calculated from the difference in the setups without and with SOD and expressed as % of control.

### 2.8. Akt1 and eNOS Protein Levels by SDS-Page and Western Blotting

HAEC cells were grown in 6-well plates and exposed to apocynin (100 µM), L-NNA (100 µM), the tested compounds (1 µM), or vehicle (MeOH) in phenol-free EBM-2 medium. After 2 h (Akt1) or 24 h (eNOS), treated cells were washed with ice-cold PBS and detached by using a cell scraper in lysis buffer (CelLytic M, Sigma, Gillingham, UK) supplemented with phosphatase inhibitors (PhosSTOP, Sigma, Gillingham, UK) and protease inhibitors (cOmplete™ Mini, Sigma, Gillingham, UK). Total protein content was quantified using the Bradford assay [34]. Ten micrograms of reduced and denatured proteins were separated by SDS-page as previously described [31] on 9% (Akt1) or 6% (eNOS) polyacrylamide gels. Proteins were transferred onto nitrocellulose membranes (Santa Cruz Biotechnology, Dallas, TX, USA), and membranes were blocked with TTBS (Tris/HCl, pH 7.5, 100 mM NaCl, 0.1% Tween 20) containing 5% BSA for 30 min at room temperature. Excess BSA was removed by washing twice with TTBS for 5 min. NOS3 (rabbit polyclonal anti-tot eNOS, cod. sc-8311, final concentration, 1:500), p-NOS (goat polyclonal anti-p-eNOS, ser 1177, cod. sc-12972, final concentration, 1:500) p-Akt1 (mouse monoclonal anti-p-Akt1, Ser473, cod. sc-293125, final concentration, 1:500), total Akt1 (mouse monoclonal anti-Akt1, B-1, cod. sc-5298, final concentration, 1:500) β-Actin (mouse monoclonal anti- β-Actin, C4, cod. Sc-47778, final concentration, 1:500) were added to the membranes in TTBS containing 1% BSA (dilution, 1:1000) and kept overnight at 4 °C. All primary antibodies were from Santa Cruz Biotechnology (Dallas, TX, USA). Membranes were washed two times with TTBS before incubation with the corresponding secondary fluorescent antibody (800CW or 680FD, Li-Cor, Lincoln, NE, USA) diluted in TTBS containing 1% BSA for 1 h at room temperature. The membrane was washed again twice with TTBS and once with TBS. The bands were visualized using the Odyssey^®^ Fc Imaging System (Li-Cor, Lincoln, NE, USA). Images were taken, processed, and quantified using the Image Studio Software (Li-Cor, Lincoln, NE, USA).

### 2.9. Statistical Analysis

Data are expressed as means ± SEM. Statistical analysis was performed using the GraphPad Prism version 7.0 software. MTT data were entered using a grouped analysis format and were analyzed by 2-way ANOVA, followed by Tukey’s multiple comparisons test with a confidence level of 95%, to assess the effect of treatments at the stated concentrations. All other results were analyzed by one-way ANOVA followed by Tukey’s multiple comparisons test with a confidence level of 95%. Significance level was set at P < 0.05.

## 3. Results

### 3.1. Cell Viability

**Figure 2:**
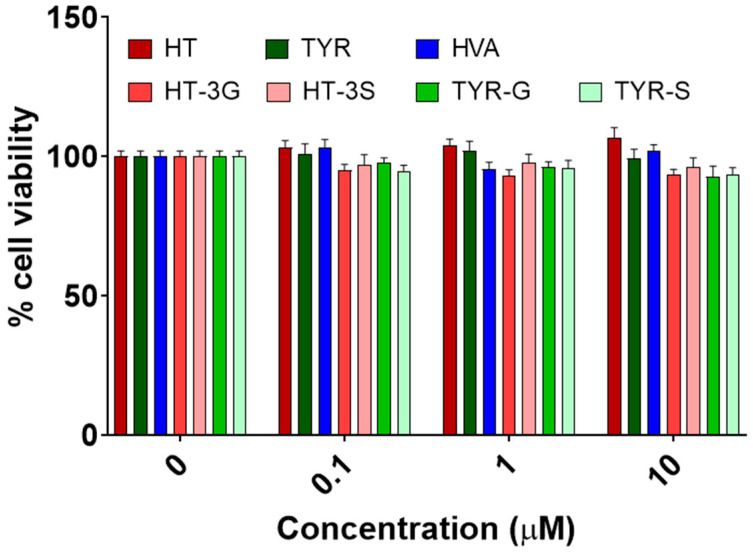
Cell viability, expressed as % of control values (100%), measured with the MTT assay, in HAEC cells exposed to EVOO phenolics and their metabolites (0.1, 1, and 10 µM) for 24 h. HT = hydroxytyrosol; TYR = tyrosol; HVA = homovanillyl alcohol; HT-3G = hydroxytyrosol-3-glucuronide, HT-3S = hydroxytyrosol-3-sulfate, TYR-G = tyrosol-glucuronide; TYR-S = tyrosol-sulfate. Data were obtained from 4 independent experiments, each performed in triplicates, and presented as average ± SEM. P > 0.05 vs. control (N = 12).

Cell viability was assessed in HAEC cultures in order to evaluate the possible cytotoxic effects of the compounds tested. The reduction of the tetrazolium dye MTT 3-(4,5-dimethylthiazol-2-yl)-2,5-diphenyltetrazolium bromide to the insoluble formazan reflected the number of viable cells present. The purple color intensity of formazan was analyzed spectrophotometrically, and the cell viability was expressed as a percentage of viability compared to the control (100% viability). Figure 2 shows the percentage of HAEC viability incubated for 24 h with increasing concentrations of the olive oil phenolics and metabolites (0.1–10 µM). The compounds did not significantly affect cell viability at any tested concentration (P > 0.05).

### 3.2. Modulation of Endothelial NO Levels as DAF2-DA Fluorescence and Nitrite Levels

To assess endothelial NO levels, both DAF-2DA fluorescence and nitrite levels after 24 h treatment of HAEC cells with the phenolic compounds of interest were assessed.

**Figure 3:**
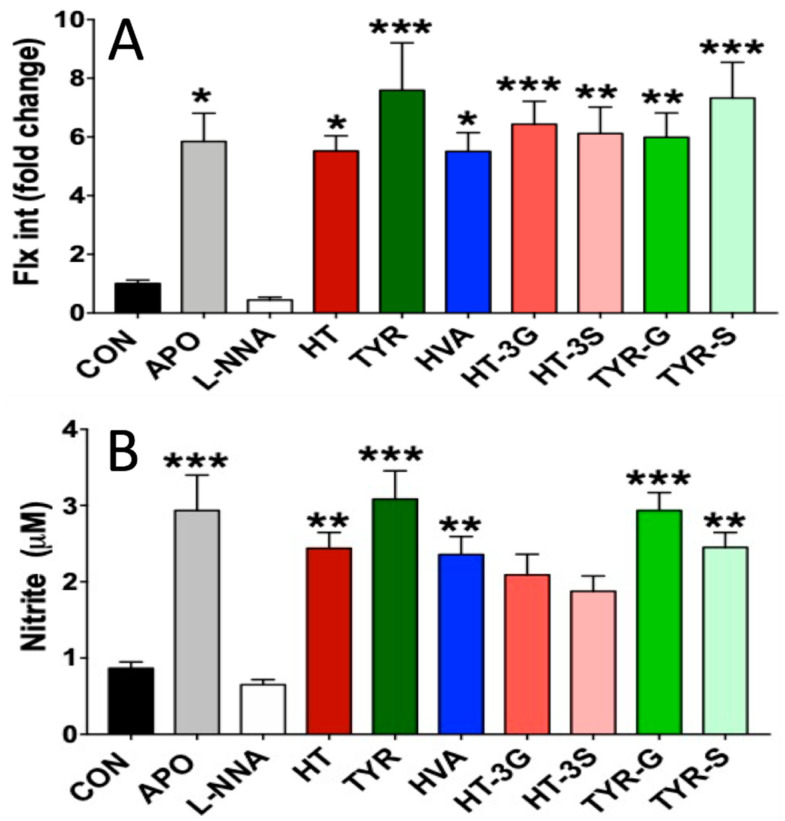
NO levels, measured as DAF-2DA fluorescence (**A**), and Nitrite levels (**B**) in HAEC cells exposed to EVOO phenolics and metabolites (1 µM), apocynin (100 µM), or L-NNA (100 µM) for 24 h. CON = control; APO = apocynin; L-NNA = L-Nω-nitro-arginine; HT = hydroxytyrosol; TYR = tyrosol; HVA = homovanillyl alcohol; HT-3G = hydroxytyrosol-3-glucuronide, HT-3S = hydroxytyrosol-3-sulfate, TYR-G = tyrosol-glucuronide; TYR-S = tyrosol-sulfate. Data were obtained from 4 independent experiments, each performed in triplicates, and presented as average ± SEM. P > 0.05 vs. control (N = 12). * P < 0.05, ** P < 0.01, *** P < 0.001 vs. control.

Figure 3A shows that the DAF-DA fluorescence intensity slightly decreased in cells treated with L-NNA (not significant), while they were significantly increased in cells exposed to apocynin (P < 0.05). Among EVOO polyphenols, TYR, TYR-S, and HT-3G showed the higher efficacy in increasing NO levels (<0.001), followed by HT-3S and TYR-G (P < 0.01). HT and its metabolite HVA were also able to significantly increase NO levels (P < 0.05) compared to control cells, though to a lesser extent. NO produced in endothelial cells undergoes a series of reactions to produce nitrite as a more stable breakdown product. Figure 3B indicates that nitrite levels were slightly decreased in cells treated with L-NNA (not significant; P > 0.05) compared to controls, whereas HT-3G and HT-3S produced a nonsignificant increase in nitrites levels in cell lysates. However, nitrite levels were significantly increased in cells exposed to APO, TYR, TYR-G, TYR-S, HT, and HVA.

### 3.3. Endothelial cGMP Levels

NO produced in endothelial cells acts through mechanisms mediated primarily via the production of cGMP [32]; therefore, the levels of cGMP in HAEC cells were assessed as an indirect measure of NO release after treatments with the compounds of interest.

**Figure 4:**
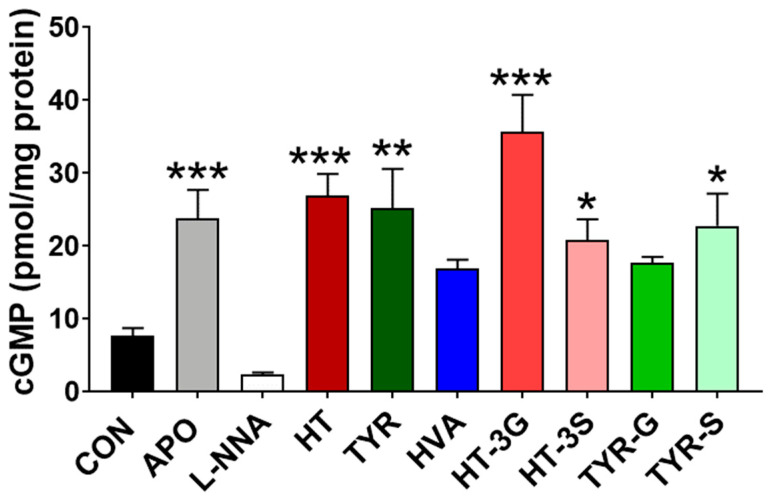
cGMP levels in HAEC cells exposed to EVOO phenolics and metabolites (1 µM), apocynin (100 µM), or L-NNA (100 µM) for 24 h. CON = control; APO = apocynin; L-NNA = L-Nω-nitro-arginine; HT = hydroxytyrosol; TYR = tyrosol; HVA = homovanillyl alcohol; HT-3G = hydroxytyrosol-3-glucuronide, HT-3S = hydroxytyrosol-3-sulfate, TYR-G = tyrosol-glucuronide; TYR-S = tyrosol-sulfate. Data were obtained from 3 independent experiments, each performed in duplicates, and presented as average ± SEM. (N = 6). * P < 0.05, ** P < 0.01, *** P < 0.001 vs. control.

Figure 4 shows that cells treated with L-NNA, HVA, and TYR-G did not produce a significantly different amount of cGMP in comparison to the control (P > 0.05). The highest levels were instead measured in cells treated with HT-3G, HT, and APO (P < 0.001), followed by TYR (P < 0.01), HT-3S, and TYR-S (P < 0.05).

### 3.4. Endothelial Superoxide Levels

**Figure 5:**
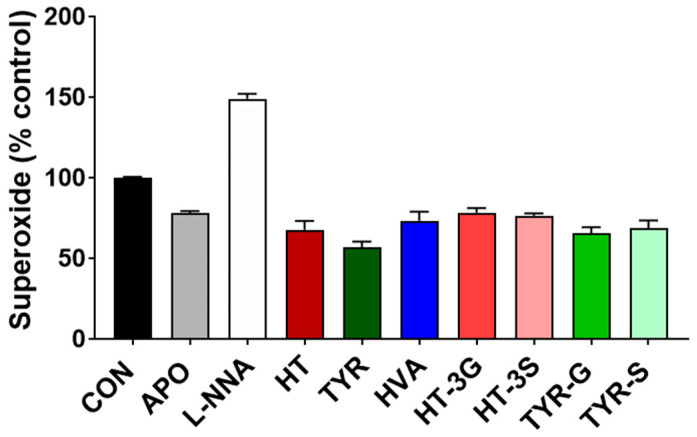
Superoxide levels in HAEC cells exposed to olive oil phenolics and metabolites (1 µM), apocynin (100 µM), or L-NNA (100 µM) for 24 h. CON = control; APO = apocynin; L-NNA = L-Nω-nitro-arginine; HT = hydroxytyrosol; TYR = tyrosol; HVA = homovanillyl alcohol; HT-3G = hydroxytyrosol-3-glucuronide, HT-3S = hydroxytyrosol-3-sulfate, TYR-G = tyrosol-glucuronide; TYR-S = tyrosol-sulfate. Data were obtained from 3 independent experiments, each performed in quadruplicates, and presented as average ± SEM. (N = 12). P < 0.001 vs. control for all groups.

The superoxide levels were measured in HAEC cells treated with the phenolic compounds for 24 h using an assay based on ferricytochrome c reduction in the presence or absence of superoxide dismutase (SOD) as previously described [31]. The results are expressed as a percentage of superoxide production of the control cells (Figure 5).

As expected, cells exposed to L-NNA were found to have significantly higher levels of superoxide compared to control cells (P < 0.001), whereas cells exposed to apocynin had significantly lower levels than control cells (P < 0.001). Similarly, the exposure of HAEC cells to the EVOO-derived phenolics and their metabolites induced a significant reduction of superoxide levels compared to control cells (P < 0.05). In particular, TYR was found to be the most effective (P < 0.001) to a level higher than apocynin.

### 3.5. Evaluation of Akt1 and eNOS Protein Levels by Western Blotting

**Figure 6:**
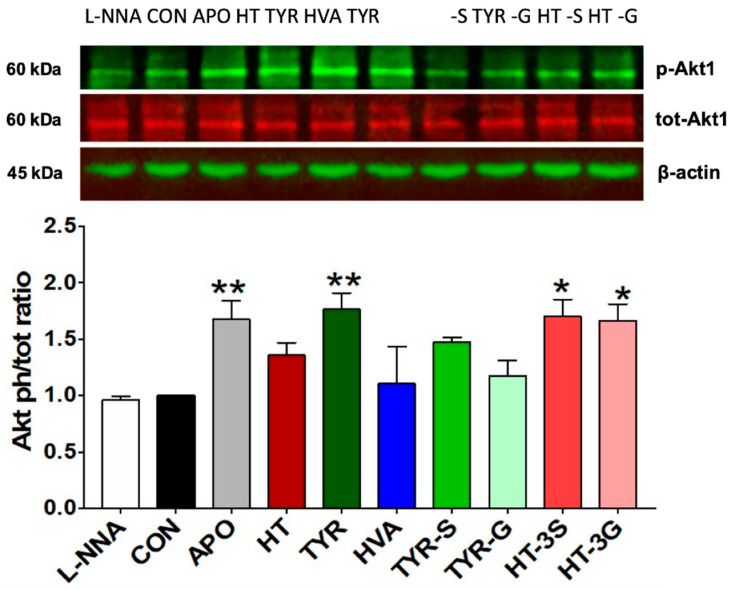
Akt1 (Ser 473) activation state, measured as phospho/total ratio, in HAEC cells exposed to EVOO phenolics and metabolites (1 µM), apocynin (100 µM), or L-NNA (100 µM) for 2 h. CON = control; APO = apocynin; L-NNA = L-Nω-nitro-arginine; HT = hydroxytyrosol; TYR = tyrosol; HVA = homovanillyl alcohol; HT-3G = hydroxytyrosol-3-glucuronide, HT-3S = hydroxytyrosol-3-sulfate, TYR-G = tyrosol-glucuronide; TYR-S = tyrosol-sulfate. Data were obtained from 6 independent experiments and presented as average ± SEM. (N = 6). * P < 0.05, ** P < 0.01 vs. control.

To investigate the potential modulation of the Akt/eNOS pathway, HAEC cells were exposed to the compounds of interest for 2 h (Akt1) or 24 h (eNOS). The protein band intensity values were normalized using the corresponding values of β-actin, and the activation state of both Akt1 and eNOS was expressed as a phosphorylated/total protein ratio. The data in Figure 6 indicates that cells exposed to APO showed a significantly higher Akt1 activation state (P < 0.01), whereas L-NNA did not induce any significant change (P > 0.05) compared to control cells. A significantly higher level of Akt1 activation was also observed for TYR (P < 0.01), HT-3S (P < 0.05), and HT-3G (P < 0.05), whereas HT, HVA, TYR-S, and TYR-G did not induce any significant change.

**Figure 7:**
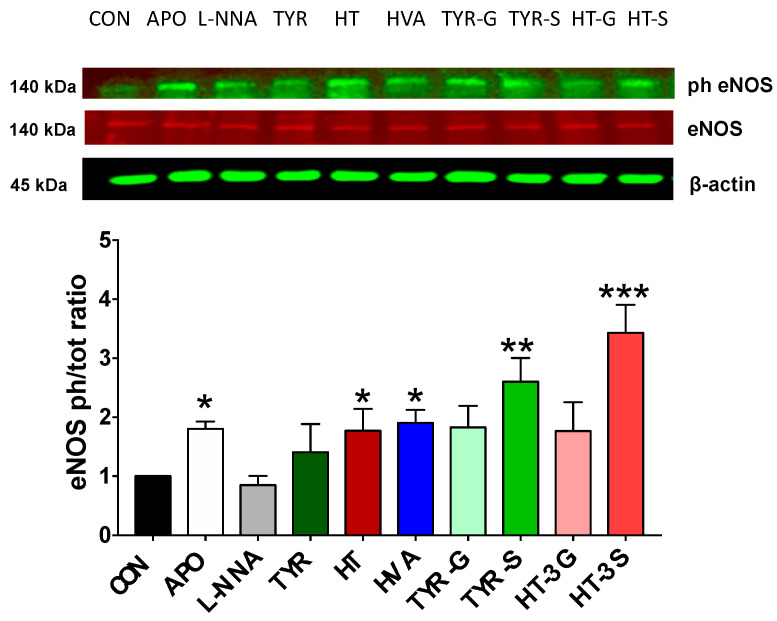
eNOS (Ser 1177) activation state, measured as phospho/total eNOS protein ratio, in HAEC cells exposed to olive oil phenolics and metabolites (1 µM), apocynin (100 µM), or L-NNA (100 µM) for 24 h. CON = control; APO = apocynin; L-NNA = L-Nω-nitro-arginine; HT = hydroxytyrosol; TYR = tyrosol; HVA = homovanillyl alcohol; HT-3G = hydroxytyrosol-3-glucuronide, HT-3S = hydroxytyrosol-3-sulfate, TYR-G = tyrosol-glucuronide; TYR-S = tyrosol-sulfate. Data were obtained from 4 independent experiments and presented as average ± SEM. (N = 4). * P < 0.05, ** P < 0.01, *** P < 0.001 vs. control.

The activation state of eNOS (Ser 1177) is reported in Figure 7, and the data indicate that the exposure of HAEC cells to APO, HT, HVA, TYR-S, and HT-3S induced a significant increase in the eNOS activation state (P < 0.05) whereas the increased activation observed after exposure to TYR, TYR-G, and HT-3G were not statistically significant (P > 0.05).

## 4. Discussion

Research studies suggest that phenolic compounds found in a range of plant foods, including EVOO, can promote endothelial function and help maintain NO availability [3,15,35,36,37], acting through oxidative-stress-related mechanisms and redox-sensitive signaling pathways [3,38]. However, the full molecular mechanisms in the endothelium are not completely clarified. The present study was designed to explore for the first time the potential of EVOO phenolic metabolites, specifically the methylated, sulfated, and glucuronidated forms of HT and TYR, to modulate NO balance in primary HAEC. These cells are derived from a human arterial endothelium and are considered a suitable cell model for evaluating mechanisms related to endothelial function and NO balance [39].

HT and TYR possess powerful antioxidant properties [17,22,40], and a range of different studies have investigated their protective/preventive effects against chronic conditions, including vascular dysfunctions [12,15], where they are shown to act not only via direct antioxidants mechanisms but also through the modulation of gene expression and intracellular signaling pathways [3,41]. Although more studies have been conducted on the parent forms of phenolic acids and most other polyphenol classes, due to the physiological relevance of their metabolic forms, recent investigations have started to highlight the potential bioactivity of both phase I and phase II metabolic forms [40,42,43], which are found to be as effective as their parent compounds [31,44]. For example, glucuronides and sulfates of parental forms can be effective modulators of cell signaling [27], platelet function [40], NO availability at the endothelial level [45], and reduce the secretion of vascular cellular adhesion molecules [44], thus accounting for the effects associated with the consumption of their parent compounds.

In our study, HAEC cells were exposed to the physiologically relevant metabolic forms of HT and TYR, namely HVA, HT-3G, HT-3S, TYR-G, and TYR-S, as well as the parental forms, at a nontoxic (as indicated by the MTT test) and physiologically relevant concentration of 1 µM, which can likely be reached in vivo in circulation after ingestion of foods containing the free forms [27,46]. The analysis of cellular NO levels carried out by using the DAF2-DA fluorescent probe indicated that apocynin and all the EVOO-derived compounds significantly increased NO levels in comparison to the control group, with TYR and TYR-S exerting the strongest effect. We additionally assessed the levels of nitrites in HAEC cells, as a more stable metabolic product derived from NO [47], and also, in this case, cells exposed to apocynin and EVOO-derived compounds had higher levels of nitrite in comparison to control cells. TYR and TYR-G were found to be the most effective in increasing nitrite concentration, whereas the nitrite increase in cells exposed to HT-3G and HT-3S was not significant in comparison to control cells.

One of the main targets in NO signaling is soluble guanylate cyclase (sGC), and its activation leads to the conversion of GTP to the intracellular signaling molecule cGMP, one of the main mediators of vasodilation [48]. Therefore, NO and cGMP production is strictly correlated, and cGMP production directly mediates the biological effects of NO [7,49]. Our data confirm this, with apocynin and most EVOO phenolics tested being able to enhance cGMP release in HAEC cells (Figure 5), while HVA and TYR-G did not significantly affect cGMP levels, although they significantly increased NO levels in comparison to control cells, suggesting the potential involvement of additional downstream mechanisms involved in the regulation of cGMP balance/degradation, which could potentially affect the latency of its vasodilatory effect [50] Furthermore, the endothelial levels of NO and consequently of cGMP may also be adversely affected by the activity of NADPH oxidase [50] through the production of superoxide, which in turn can quickly react with NO reducing its availability with the formation of peroxynitrite, followed by spontaneous isomerization to nitrate or oxidation and nitration of biomolecules [50]. Apocynin is a known inhibitor of NADPH oxidase, and our data show that in accordance with this mechanism, apocynin is able to significantly reduce superoxide formation in HAEC cells. In this study, the release of superoxide in HAEC cells after exposure to the EVOO-derived phenolic compounds was reduced, thus leading to an increase in the cellular NO balance. These compounds can potentially exert this effect on superoxide suppression in different ways through both direct scavenging properties, as well as NADPH oxidase inhibition. Indeed, both HT and TYR are powerful antioxidant compounds [16,51], and therefore, they are able to decrease superoxide levels by acting as primary antioxidants. In addition, these phenolic acids possess structural analogies to known NADPH oxidase inhibitors, and therefore, this potential mechanism of action cannot be discounted [52,53]. HT, for example, is shown to be able to inhibit NADPH oxidase expression and activity [54,55], in addition to its direct scavenging activity. In regard to metabolic forms, the direct antioxidant/scavenging activity of conjugated metabolites is likely to be affected by the metabolic conversion to glucuronides and/or sulfates. For example, the antioxidant properties of HT and TYR metabolites in a range of concentrations compatible with their dietary consumption were evaluated, and none of the glucuronides displayed significant antioxidant activities at physiologically relevant concentrations [56].

To further elucidate the cellular mechanism involved in the maintenance of endothelial NO balance, we also investigated the potential ability of EVOO-derived compounds and metabolites to modulate the Akt1/eNOS pathway, a known potential contributor to the maintenance of NO levels in endothelial cells [57]. Indeed, Akt1/eNOS activation can be enhanced by different stimuli and intracellular signals, and different plant-derived polyphenols have the potential to modulate the Akt1/eNOS pathway [3], as wholegrain-derived phenolic acids and their metabolites are already proven to induce Akt1 phosphorylation and increase eNOS levels in endothelial cells [31]. Herein, we observed that TYR (P < 0.01), as well as HT-3G and HT-3S (P < 0.05), was able to significantly induce Akt phosphorylation. These results are in agreement with in vivo experiments in rat models, where Akt activation by both TYR and HT was ascertained [58,59]. In addition, our data indicated that the eNOS activation state (Ser 1177) in HAEC exposed to EVOO-derived compounds was significantly higher in HAEC cells exposed to HT, HVA, TYR-S, and HT-3S, whilst the glucuronide conjugates did not induce any significant modulation of eNOS activation state, highlighting how bioactivity can be maintained after conjugation/metabolic conversion of the parental forms. The beneficial properties of TYR, TYR-G, and TYR-S against oxidative stress and inflammation in TNF-α-treated human umbilical vein endothelial cells (HUVEC) are reported in the literature, showing that TYR metabolites, particularly the sulfate form, can ameliorate the TNF-α-induced oxidative stress to a comparable/superior extent that TYR itself [60]. Similarly, HT-3S can attenuate the morphological changes induced by the inflammatory stimuli IL-1β in endothelial cells, suggesting that HT-3S might have a role in the prevention of endothelial dysfunction and related pathologies [61].

The ineffectiveness of glucuronidated compared to sulfated compounds, as in the case of TYR conjugates, has also been recently observed on endothelial cells treated with ferulic acid and its phase I/II metabolites, where the glucuronides did not elicit eNOS expression, unlike sulfates and parental compounds [31]. A dated cell culture study conducted using a somatic/endothelial cell hybrid suggested that direct modulation of eNOS levels by HT was unlikely [62]; thus, a more recent study highlighted the positive modulation of PI3K-Akt-eNOS protein pathways by HT [63]. Our results add support to the latest finding, showing a positive modulation of the PI3K-Akt-eNOS protein pathways by HT. Our study assessed the individual contribution of each metabolite tested; thus, potential higher synergistic effects could be achieved, and future studies should further explore this aspect. Furthermore, additional ex vivo/in vivo assessments would add strength to our study outcomes. It is also worth noting that the potential metabolic conversion of TYR into HT in vivo should be considered, since TYR activity may be the result of the combined action of both HT and TYR, thus influencing the assessment of intracellular TYR activity [41]. To assess the relevance of our findings in a more pathological context, future studies should aim to assess if our compounds of interest can maintain their ability to modulate NO balance in the presence of a pathological relevant stressor.

## 5. Conclusions

**Figure 8:**
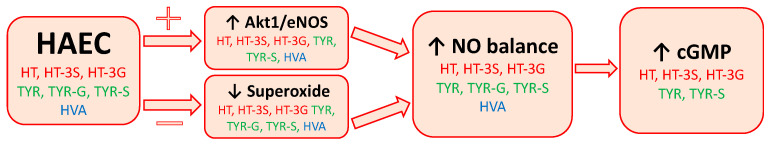
Schematic representation of the mechanisms involved in the modulation of endothelial NO levels by the EVOO-derived metabolites tested in the present study.

In summary, we show that metabolic forms of EVOO-derived phenolics, as well as their parental free forms, possess the ability to contribute to the maintenance of NO balance in primary human endothelial cells (Figure 8).

Primarily, all compounds tested were able to reduce NO depletion in HAEC by limiting superoxide cellular levels, conceivably acting through antioxidants mechanisms. The inhibition of NADPH oxidase can be assumed, but the mechanisms by which the tested phenols and their metabolites actually influence the superoxide production need to be further elucidated. Additionally, HT, HVA, HT-3S, HT-3G, and TYR-S proved to also be active in enhancing Akt1/eNOS activation. Through both positive (enhanced production) and/or negative (reduced degradation) NO levels, all metabolic forms were able to contribute to the maintenance of NO balance in HAEC cells, with a significant contribution of HT, HT-3S, HT-3G, TYR, and TYR-S to the increase in the second messenger cGMP, an effector molecule of vasodilation. All these findings therefore suggest that not only EVOO-derived phenolics parental free forms but also their major in vivo formed metabolites may exert positive effects on endothelial NO balance and homeostasis.

## Sample Availability

The samples are commercially available.
